# Saiga horn user characteristics, motivations, and purchasing behaviour in Singapore

**DOI:** 10.1371/journal.pone.0222038

**Published:** 2019-09-10

**Authors:** Hunter Doughty, Diogo Veríssimo, Regina Chun Qi Tan, Janice Ser Huay Lee, L Roman Carrasco, Kathryn Oliver, E. J. Milner-Gulland

**Affiliations:** 1 Department of Zoology, University of Oxford, Oxford, United Kingdom; 2 Institute for Conservation Research, San Diego Zoo, Escondido, United States of America; 3 Singapore, Singapore; 4 Asian School of the Environment, Nanyang Technological University of Singapore, Singapore; 5 Department of Biological Science, National University of Singapore, Singapore; 6 Faculty of Public Health and Policy, London School of Hygiene and Tropical Medicine, London, United Kingdom; University of Bucharest, ROMANIA

## Abstract

Unsustainable wildlife trade is a pervasive issue affecting wildlife globally. To address this issue, a plethora of demand reduction efforts have been carried out. These necessitate consumer research which provides crucial knowledge for designing and evaluating targeted interventions. We implemented a rigorous consumer survey on saiga (*Saiga tatarica*) horn use in Singapore, where usage is legal and widely sold. Saiga are Critically Endangered antelopes from Central Asia with horns (often marketed as *ling yang*) used in traditional Chinese medicine (TCM). Few past studies have assessed saiga horn consumers. This work is the most extensive consumer research to date specifically characterising saiga horn consumers and usage. We conducted 2294 in-person surveys on saiga horn use with Chinese Singaporeans, employing neutral questioning approaches. We found 19% of individuals reported saiga horn as a product they choose *most often* for themselves and/or others when treating fever and/or heatiness (a TCM state of illness), indicating a minimum estimate of high-frequency usage, not including possible low-frequency users. Overall saiga users were most characterised as middle-aged Buddhists and Taoists. However, saiga users were found in a range of demographic groups. Women preferred saiga shavings (the more traditional form), while men preferred saiga cooling water (the more modern form). About 53% of individuals who used saiga horn themselves also bought it for someone else. Buyers for others were most likely to be female middle-aged Buddhists or Taoists. Key motivating reasons for usage were “it works” and “someone recommended it to me.” The top two reported recommenders were family and TCM shopkeepers. Saiga users were more likely than non-saiga users to perceive saiga as a common species in the wild. This research holds significance for interventions targeting saiga horn consumption within Singapore and throughout Asia, by identifying potential target audiences, product types, non-desirable alternatives, and motivations for use.

## Introduction

Goods made from wild animals are used pervasively across the globe for consumptive, medicinal, and cultural purposes. The scope of trade across the multitude of species used includes both legal and illegal markets, as well as sustainable and unsustainable levels of demand [1]. When consumer demand has become too large for a species to sustain, it often causes drastic population declines, which has driven species of a wide taxonomic range towards extinction [2]. In addition to ecological impacts, the international transport of wild animals and their parts has been linked to the spread of several diseases, including the 2003 SARS pandemic [3], and illegal wildlife trade specifically has been cited as a threat to local livelihoods and safety [4,5].

Illegal and/or unsustainable wildlife trade is a multi-faceted issue that requires a multi-pronged solution with demand reduction as a vital prerequisite for long-term success [2,5,6]. Behavioural interventions carried out primarily by non-government organisations (NGOs) have thus multiplied rapidly across the globe, especially in Asia [7]. To implement a behavioural intervention, however, requires a detailed understanding of the consumers the intervention is targeting. In the fields of public health and social marketing, where behavioural interventions have been extensively implemented for decades, this implies rigorously collected datasets, that are defensibly illustrative of the consumer landscape [8,9]. In recent years those working in illegal and/or unsustainable wildlife trade have acknowledged shortcomings in the design, implementation, and evaluation of behavioural interventions targeting wildlife consumers [10,11]. Particular criticism has been drawn to the validity of data collection methods and the findings extrapolated from these datasets [12,7]. Similar points have been made across conservation, when assessing the social studies that are often used to inform or evaluate human behaviour interventions [13]. These methodological shortcomings likely compromise researchers' capacity to assess usage of critically endangered species impacted by unsustainable trade.

One species greatly affected by human demand is the saiga antelope (*Saiga tatarica*). Saigas are Critically Endangered antelopes from Central Asia [14]. The species' horn (often marketed as *ling yang* (羚羊)) is used in many traditional Chinese medicines (TCM), and the species has suffered a major decline (>95%) in the 1990s due to poaching for horn and meat [15,16]. Despite a popular misconception that this increase in poaching was driven by conservationists recommending saiga horn as a replacement for rhino horn, demand for saiga horn in TCM has existed for centuries, and this particular rapid increase in poaching was in fact a direct result of the collapse of the Soviet Union and the subsequent loss of law enforcement and management capacity in the saiga's range states, as well as rampant rural poverty [15,16]. In addition to these threats, recent mass bacterial and viral disease-induced die-offs have further substantially reduced saiga’s population levels [17]. To help curb poaching pressure, international trade in saiga products has been regulated by the Convention on International Trade in Endangered Species of Wild Flora and Fauna (CITES) since it was added as an Appendix II listed species in 1995 [18]. Additionally, the species has a Memorandum of Understanding under the Convention on Migratory Species covering conservation actions [19]. Thus, there is limited legal trade between consumer countries, and all the range states have voluntary moratoriums on export of saiga products. Despite these many policy efforts though, poaching of saiga still persists [19].

Singapore is recognised as one of the largest saiga horn consumer countries, and sales of saiga horn and its derivatives within Singapore are permitted if they are pre-Convention stock or have been legally imported with CITES permits [20]. Therefore, saiga products are widely available in shops and their purchase is non-sensitive. A 2016 small-scale survey of saiga product usage in Singapore found 13% of their sample of 230 Chinese Singaporeans were current users of saiga products, and that saiga horn products were sold throughout Singapore, in the form of shavings, ground up in saiga cooling water or medicinal pills, or intact as whole horns [21]. Without a large-scale study though, a robust estimate of usage has not been known. Saiga horn is most commonly used to treat fever or heatiness (a TCM state of illness with symptoms such as sore throat, nasal congestion, and cough). In TCM, Singapore is considered a heaty and damp cityscape [22], and thus many Singaporeans ingest cooling substances often. These can be specific medicines or broths prescribed by TCM practitioners or recommended by TCM shopkeepers (with ingredients such as saiga horn, chrysanthemum, and honeysuckle), or they can be simply certain kinds of foods that are considered cooling such as citrus fruit, tofu, and cream.

To address the timely need for detailed research on saiga horn consumers, this work aims to understand consumption patterns of medicines containing saiga horn in Singapore, through the most extensive and robust study of a saiga horn consumer population to date. By assessing:

- prevalence of use,
- socio-demographic characteristics of saiga horn users,
- purchase trends (i.e. who buys for others and what additional treatment types consumers use),
- stated reasons for use,
- and conservation awareness,

we provide insight into potential target audiences, product types, and intervention angles for a behavioural intervention design to reduce the use of saiga products in Singapore. We thus hope this research will prove a sound foundation for any future demand reduction efforts with these consumers. We furthermore aimed to implement a robust approach to consumer survey design and implementation, that may be useful for other researchers.

## Methods

### Survey overview

The survey focused on asking participants about their preferred treatment for fever and heatiness in themselves and others (please see S1 Table for the full survey). The first section assessed heatiness preferences for themselves and then others (if they indicated they buy treatments for others), followed by fever preferences, a single question assessing their perception of the abundance of wild populations of a set of species used in TCM, and finally the demographic questions. The format and questions were a result of iterative drafts and scrutiny by the authors, informed by discussions with the relevant government agencies (the Agri-Food & Veterinary Authority of Singapore (now under the National Parks Board of Singapore) and the Health Science Authority of Singapore), as well as Wildlife Reserves Singapore. The survey was piloted for two days (~75 surveys) with members of the public at the National University of Singapore and in a Planning Area not used in our study (Singapore is divided into 56 Planning Areas as demarcated in the Urban Redevelopment Authority’s Master Plan 2014 [23]). Feedback from the pilots helped to refine question phrasing and formatting. For example, we found that the heatiness section had a higher cognitive burden than the fever section (i.e. it required more thought on the part of the respondent), and thus we placed the heatiness section first so that respondents could answer it while they were freshly engaged.

The following sections about survey questions and sampling highlight our efforts to employ neutral questioning approaches. Each of these components was used to mitigate the probability of potential biases, including social desirability bias (i.e. that we would inadvertently prompt our respondents to inflate or deflate their saiga horn consumption depending on what they thought we wanted to hear), thereby increasing the probability of obtaining accurate figures for saiga product usage.

### Question format

Questions were organised into a decision-tree style format, allowing respondents to answer general questions first, and only be asked further questions as they were applicable to them. The top-level question for fever, for instance, simply asked which treatment types (western, traditional herbal, traditional animal, or other) that they used most often. We used the term western medicine in our survey to refer to biomedical medicine, as this was the phrase determined to be the most commonly understood by our target audience based on our scoping trip and discussions with relevant in-country stakeholders. If a respondent stated that they use western and other treatments in the survey, then they would be asked about types of western and other treatments, but they would not answer any questions on traditional treatments. This approach to questioning allowed us to not ask about saiga product usage directly, but rather have it as an option for those who selected traditional animal products in these top-level questions. Those who selected saiga horn at this point could then answer further questions regarding their usage, such as product form and purchase location.

### Question phrasing

Questions were framed as neutrally as possible so as not to prompt respondents into choosing one answer over another. If for example, a respondent had stated in a previous question that they purchased traditional animal products when treating a teenager with heatiness, then the next question was “When treating a teenager's heatiness with traditional animal products: which type of animal products do you use most often? [Select all that apply],” followed by other questions such as “What form of treatment do you use most often?”. We specifically asked which health treatments respondents use *most often* in the given circumstance as that would give us a robust minimum estimate of the prevalence of use. However, it does not necessarily capture all users who may not consider saiga horn a product they use most often, but whom do consume it at times. A respondent's ability to select multiple choices as their most used health treatments aimed to mitigate this limitation.

### Target audience, location, and timing

Using 13% as an estimate of the percentage of saiga product users among Chinese Singaporeans (based on a prior small-scale study [21]) we assessed the sample size we would need to have statistical power when running analyses with the user sub-samples. Using nested analyses as an initial gauge, we conducted a power calculation for performing a three-tiered nested proportion statistical test with three of our main questions (S1 File). This calculation estimated that we would need a minimum of ~1500 respondents. We then based our sampling plan on the co-authors' experiences of past survey recruitment rates along with rates from the pilot days. However, the actual recruitment rate during our survey was higher than anticipated, and thus we were able to collect more surveys.

Many ethnicities in Singapore have been cited as being TCM users, but Chinese Singaporeans make-up the largest ethnic group (84%) of complementary and alternative medicine users (inclusive of TCM) [24]. We therefore chose to focus on them. Consumer surveys were conducted in the Planning Areas with the top five largest populations of Chinese Singaporeans (S2 Table). Within each Planning Area, we identified four locations that attracted people of varying socio-economic levels including malls with high-end or lower-end stores, hawker food centres with affordable open-air food stalls, and generally popular areas with restaurants and shops (S2 Table). Survey numbers across Planning Areas were distributed proportionally to Planning Area Chinese Singaporean population. Surveys were also stratified across day of the week and time of day (weekday/weekend and morning-afternoon/afternoon-evening shifts) to capture as many different types of people with varying schedules as we could. To sample evenly across gender and age groups (18–35 years, 36–59 years, ≥ 60 years), we checked the demographic characteristics of the respondent sample weekly. Distributions were naturally at most 3% different for age, and under 8% for gender, but to gain more gender balance, in the last day we only sampled women, with all other collection protocols remaining the same.

### Sampling method

There are a number of sampling methods that could be employed to conduct consumer surveys. For example, they could have been conducted online through the use of a third-party survey platform [25], or via snowball sampling using contacts to build-up a respondent pool [26]. We chose intercept surveys in which researchers intercepted individuals in-person in public places outside of our chosen locations. These allowed us to engage independently with different members of the general population from a diverse range of backgrounds, who were not incentivised by compensation for participation, had no affiliations with our networks, and were not required to have internet access.

All four survey researchers were in their early twenties, female, and Chinese Singaporean. Surveys were designed and managed using Open Data Kit, and administered using tablets. Respondents who did not wish to use the tablet themselves had the choice to have the researcher ask them questions orally. The survey was available in both English and Mandarin, and the researchers were fluent in both languages.

Researchers wore identifiable Nanyang Technological University (NTU) logo shirts so as to not be mistaken for saleswomen. Surveys were described to potential participants as assessing heatiness and fever treatment preferences, with no mention of saiga, TCM, or wildlife. This was in order not to prime participants into pre-emptively thinking about these topics, or into thinking that we were especially interested in these topics (which could lead to social desirability bias). Every third person who passed a researcher (while the researcher was not with another respondent) was asked to participate. If a group of people approached, the researcher asked the person on the right (for a group of two) or the third person to the right (for groups larger than two). It is possible that there is some bias in certain types of people always being on one side of a group, however, we hope that through our balanced collection of gender and age types we have mitigated for this. Only the chosen person in a group was allowed to take the survey.

### Data analysis

**Fig 1:**
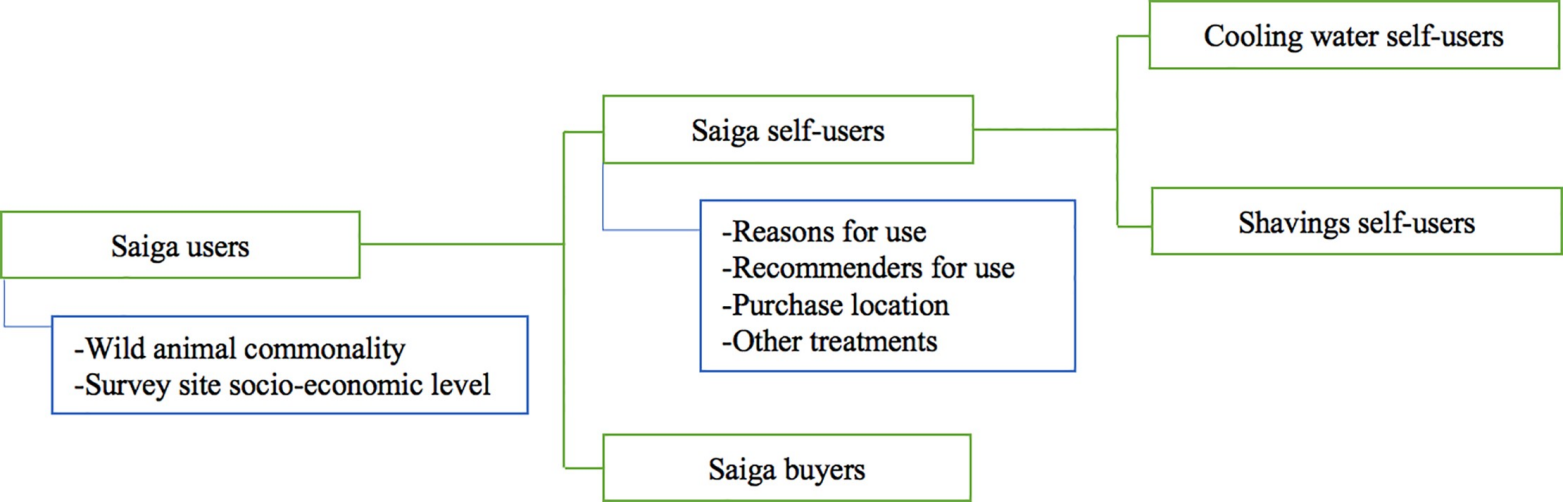
Data analysis breakdown. Fig 1. Data analysis breakdown showing saiga horn sub-user groups, assessed using averaged GLMs with contrasts, along with the additional questions that are asked of two groups of particular interest. All respondents who use and/or buy saiga horn for themselves and/or others are considered "saiga users". Respondents who use it themselves are considered "saiga self-users", and those who buy for others are "saiga buyers" (self-users and buyers are not mutually exclusive, and both are subsets of users). Within saiga self-users, we also characterised respondents who stated that the saiga products they purchase most often are cooling water or shavings.

The data were first assessed through descriptive and visual analyses, followed by a statistical analysis using R 3.5.2 GUI 1.70 (7612 El Capitan build). We preferred parametric tests over non-parametric tests because of their greater statistical power, and because parametric tests can be used even when groups have different levels of variability (as was the case with our data) [27]. We first evaluated whether the datasets met the assumptions of parametric tests using visual and frequency-based analyses. Demographic data analysis for various user sub-groups (Fig 1) began with a summary of each demographic variable (Table 1). Data were not weighted because we were interested in identifying potential target demographic groups for future interventions, rather than producing a population-wide estimate of product consumption levels. At this stage though, sub-levels of some variables with low (<10) or no counts were combined, for example uncommon religious affiliations were all combined into “Other religion”. Then, generalised linear models (GLMs) were used to explore associations between those belonging to a saiga horn user sub-group, and other dependent variables (Table 1). The initial GLM outputs led us to choose model averaging, in order to minimise researcher bias in model selection as there were no clear top models. We used the package MuMim [28], included all seven demographic variables, and averaged models with an Akaike information criterion (AIC) < 4.

**Table 1:** Demographic variables used in regression analyses, along with their *a priori* hypothesised effects in each consumer sub-group.

Variable	Type	Consumer Group	Hypothesis ^	
			Variable Effect	Direction of Effect
Age	Ordinal	Total saiga users, self-users, & buyers	Yes	Older age more likely to use or buy ^[^^29^^], a, b^
		Cooling water self-users		Younger age more likely to use ^[^^29^^], a, b^
		Shavings self-users		Older age more likely to use ^[^^29^^], a, b^
Dialect	Categorical	All consumer sub-groups	No	No difference between dialects ^[^^30^^], a, b^
Education	Ordinal	All consumer sub-groups	Yes	Lower education more likely to use or buy ^[^^29^^], a, b^
Sex	Binary	Total saiga users, & self-users	No	No difference between sexes ^a, b^
		Saiga buyers	Yes	Women more likely to buy ^[^^31^^], a^
		Cooling water self-users		Males more likely to use ^[^^29^^], a^
		Shavings self-users		Females more likely to use ^a^
Generation Singaporean ^^	Ordinal	Total saiga users, self-users, & buyers	Yes	Less time that family has lived in Singapore, more likely to use ^[^^22^^], a^
		Cooling water self-users		More time that family has lived in Singapore, more likely to use ^[^^22^^], a^
		Shavings self-users		Less time that family has lived in Singapore, more likely to use ^[^^22^^], a^
Income	Ordinal	All consumer sub-groups	Yes	Lower income more likely to use ^[^^24^^], [^^29^^], a^
Religion	Categorical	All consumer sub-groups	Yes	Buddhists and Taoists more likely to use ^[^^22^^], [^^32^^], a^

^ Hypotheses were based on: general readings of TCM trends in Singapore, as well as discussions within the research team and colleagues in Singapore (denoted with symbol “a”), and a scoping trip in February 2017 (denoted with symbol “b”).

^^ Generation Singaporean is the number of generations a respondent’s family has lived in Singapore.

In conjunction with this process, we applied sum contrasts. Sum contrasts allow for a comparison of the mean of one variable level to the mean of all means for all the levels within that variable [33]. This is different from the default GLM output which compares each level to a single reference level within that variable.

The *a priori* hypothesised direction of effect for each demographic variable on the consumer sub-groups were based on insights from previous studies and from our own scoping research (Table 1). This included discussions within our team (a group of Singaporean and international researchers from varying disciplines) and with external colleagues in Singapore, along with visits to TCM shops and discussions with relevant stakeholders such as shopkeepers and government officials. Literature on TCM use in Singapore also informed our choice of variables to test. For instance, findings on spousal influence on family purchase behaviour [31] suggested women were more likely to purchase these treatments for others, and a study on TCM shop patronage [29] augmented general perceptions in Singapore that older individuals might be more likely to purchase minor ailment treatments like saiga horn in a TCM shop. That same study found that gender, education, and income were also correlated with visiting TCM shops for minor ailment treatments. An anthropological study on TCM cooling treatments in Singapore [22] and a broader text on TCM foundations [32] led us to hypothesize that Buddhists and Taoists, along with those who have more recent family ties to mainland China, would be more likely to be saiga horn users given the cultural overlaps with TCM.

To understand saiga product consumption, we analysed preferences specifically of those who use saiga products to treat themselves (saiga self-users), as these individuals are likely to present the strongest preferences. These individuals may or may not also be buyers (those who buy saiga products for others). In a preliminary analysis, preferences for the buyer group resembled the self-user group, so a second analysis of that group was not performed. Potential other treatment types preferred by saiga self-users were assessed via a GLM, while reasons for using saiga products, who recommended saiga product use, and purchase locations of saiga products, were all analysed using 2-sample z-tests for equality of proportions with a continuity correction (which was chosen because the respondent’s answers on these questions were not mutually exclusive).

Insight into the perceptions of those who purchase saiga products either for themselves or others (saiga users), was also of interest. In particular, we wondered whether being a saiga user meant that the individual was more likely to think saiga was a common species, as an indicator for conservation awareness regarding saiga. To answer this query, respondents were given a list of species that are found in TCM and asked to select the ones that they think are common in the wild. We chose to not ask about rarity as we did not want to prompt respondents into thinking that some of the given species must be rare. Results were analysed for each species via Pearson's chi-squared test for independence, and overall propensity for perceiving commonness of species was analysed via a GLM. Lastly, we used the locations in which saiga users were surveyed as a second means of assessing whether socio-demographic level was associated with saiga product use. Results were similarly analysed using Pearson's chi-squared test for independence.

### Ethics

This research was approved by the Institutional Review Board of NTU (IRB-2017-04-018) and the Central University Research Ethics Committee of the University of Oxford (R50787/RE001). Informed consent was obtained orally and recorded by the surveyors via the tablet when initiating the survey; this process was approved by the preceding review boards.

## Results

In total, 2294 surveys were conducted over a six-week period (June-July 2017) (see S3 Table for raw data). The sample was fairly balanced across age (young 34%, mid-age 34%, older 31%) and sex (male 49%, females 51%). Of these respondents, 438 (19%) were saiga users–defined as those stating saiga horn was a product they use *most often* to treat heatiness and/or fever in themselves and/or others. We found that 53% of individuals who buy saiga products for themselves also buy it for someone else. Among buyers, 84% also use it themselves.

### Consumer groups

Of the seven demographic variables assessed, age and religion had the greatest relative importance for almost all of the averaged GLM models (Table 2). Sex was also an important determinant of use for the buyer, cooling water, and shavings sub-groups.

**Table 2:** Combined model selection table for all consumer sub-groups, showing dredge models with a delta <4, and the Relative Importance (RI) of the variables in each averaged-model.

		Religion	Age	Gender	Education	Income	Generation Singaporean	Dialect	Delta	Weight
Total users	RI	1.0	1.0	0.33	0.69	0.22				
		+	+		+				0	0.319
		+	+						0.91	0.202
		+	+	+	+				1.52	0.149
		+	+		+	+			1.58	0.145
		+	+	+					2.15	0.109
		+	+	+	+	+			2.89	0.075
Self-users	RI	1.0	0.95	0.22	0.74	0.07		0.08		
		+	+		+				0	0.384
		+	+						1.38	0.192
		+	+	+	+				1.9	0.149
		+	+		+			+	3.07	0.083
		+	+	+					3.37	0.071
		+	+		+	+			3.49	0.067
		+			+				3.9	0.055
Buyers	RI	1.0	1.0	1.0		0.82	0.1			
		+	+	+		+			0	0.725
		+	+	+					2.84	0.175
		+	+	+		+	+		3.97	0.1
Cooling water self-users	RI	1.0	0.65	1.0			0.23			
		+	+	+					0	0.498
		+		+					1.19	0.275
		+	+	+			+		2.34	0.154
		+		+			+		3.83	0.073
Shavings self-users	RI	1.0	1.0	1.0	0.65					
		+	+	+	+				0	0.651
		+	+	+					1.24	0.349

Saiga users were significantly more likely to identify as Buddhist (p<0.001) or Taoist (p<0.01), and to be middle-aged (p<0.01). They were significantly less likely to be under 36 years old (p<0.05) (Table 3). The demographic characteristics of saiga users were quite variable, especially when comparing between age groups. For example, the most common education levels for younger users was pre-university or university (81%), for mid-aged users it was secondary school or the equivalent (34%), and for older users it was primary school or under (51%).

**Table 3:** The full averaged-model coefficients of variables with significant p-values, shown for each consumer sub-group.

		ß	Std. Error	Z-value	P-value ^
Total users	(Intercept)	-1.708	0.151	11.357	***
	Young age	-0.238	0.106	2.248	*
	Mid-age	0.230	0.076	3.015	**
	Old	0.007	0.113	0.063	
	Buddhist	0.547	0.145	3.761	***
	Taoist	0.613	0.219	2.792	**
	Catholic	-0.512	0.280	1.827	.
	Christian	-0.031	0.185	0.167	
	No religion	0.083	0.161	0.514	
	Other religion	-0.866	0.635	1.362	
Self-users	(Intercept)	-1.830	0.140	13.103	***
	Young age	-0.193	0.118	1.636	
	Mid-age	0.194	0.090	2.162	*
	Old	-0.001	0.115	0.004	
	Buddhist	0.603	0.148	4.061	***
	Taoist	0.458	0.232	1.969	*
	Catholic	-0.652	0.310	2.104	*
	Christian	-0.039	0.193	0.200	
	No religion	0.108	0.166	0.653	
	Other religion	-0.751	0.636	1.179	
Buyers	(Intercept)	-2.802	0.233	12.034	***
	Young age	-0.644	0.127	5.080	***
	Mid-age	0.533	0.099	5.381	***
	Old	0.111	0.115	0.963	
	Buddhist	0.432	0.202	2.140	*
	Taoist	1.037	0.275	3.773	***
	Catholic	-0.387	0.362	1.069	
	Christian	0.201	0.240	0.840	
	No religion	0.161	0.226	0.712	
	Other religion	-0.706	0.888	0.795	
	Female	0.621	0.153	4.062	***
Cooling water self-users	(Intercept)	-2.764	0.196	14.115	***
	Buddhist	0.615	0.194	3.168	**
	Taoist	0.468	0.298	1.569	
	Catholic	-0.282	0.355	0.794	
	Christian	0.043	0.245	0.175	
	No religion	0.188	0.216	0.873	
	Other religion	-0.776	0.879	0.882	
	Male	0.460	0.143	3.22	**
Shavings self-users	(Intercept)	-5.623	75.730	0.074	
	Young age	-0.595	0.207	2.875	**
	Mid-age	0.390	0.130	3.008	**
	Old	0.206	0.191	1.077	
	Female	0.867	0.198	4.371	***

^ Significance codes:

‘***’ 0.001

‘**’ 0.01

‘*’ 0.05 ‘.’ 0.1

We saw similar demographic characteristics between the self-user (403 respondents– 18%), and buyer (223 respondents– 10%) groups. Females were more likely than males to buy saiga products for others, as were Buddhists and Taoists, and middle-aged individuals; young people were less likely to buy saiga products for others (all p<0.001). Those who buy saiga products for others were similar to those who buy any treatment type for others in terms of sex and age, but buyers of any treatment type for others were more likely to have a higher educational level than non-buyers, and religion did not affect their buying decisions (S2 File).

There were product-specific differences in self-usage when assessing the two most cited forms of saiga product: cooling water (54% of self-users) and shavings (33%). Cooling water self-users totalled 231 respondents (10%), and were more likely to be male (p<0.01). Shavings self-users totalled 138 respondents (6%), and were most likely middle-aged (p<0.01), and female (p<0.001). Cooling water was preferred by 66% of young self-users, 59% of middle-aged, and 49% of older self-users. An almost opposite trend was seen for shavings: 19% of young self-users preferred shavings, 39% of middle-aged, and 39% of older self-users.

### Self-user preferences

**Fig 2:**
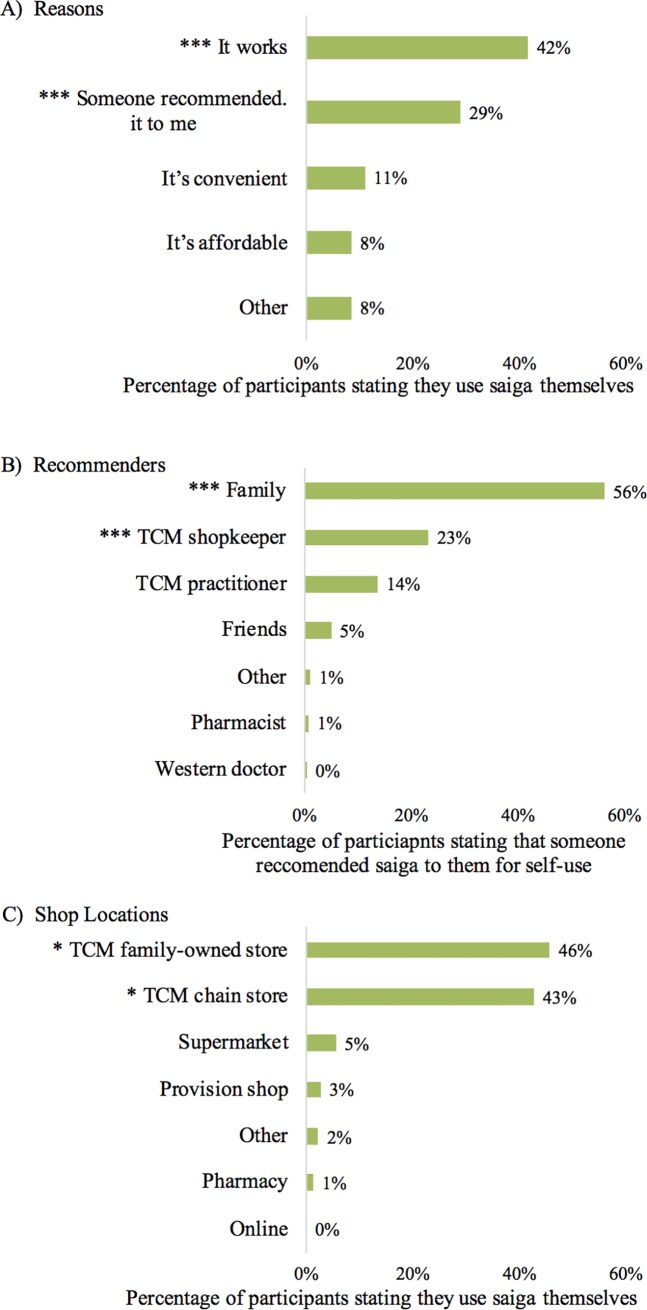
Self-user motivations and preferences. Asterisks indicate a statistically significant difference between this answer and all others within a panel (2-sample Z-tests, p-value < ‘***’0.001 or ‘*’ 0.05). A) Reasons for using saiga products on oneself to treat heatiness and/or fever, as reported by participants. Percentage out of 403 participants. B) Recommenders for using saiga products on oneself to treat heatiness and/or fever, as reported by participants. Percentage out of 186 participants. C) Shop locations for purchasing saiga products to treat heatiness and/or fever on oneself, as reported by participants. Percentage out of 403 participants.

Motivations and preferences of self-users provide important insights into product use. When self-users were asked why they preferred saiga products to treat heatiness and/or fever, they reported "It works" (42%) and "Someone recommended it to me" (29%) more often than other reasons (χ^2^(1, *N* = 403) = 33.09, *p* <0.001; χ^2^(1, *N* = 403) = 74.24, *p* <0.001 respectively; Fig 2A). For self-users who stated that saiga was recommended to them, "Family" (56%) and "TCM shopkeeper" (23%) were reported more often than other options (χ^2^(1, N = 186) = 97.51, p <0.001; χ^2^(1, N = 186) = 8.97, p <0.001 respectively; Fig 2B).

Self-users purchased saiga products far more often in TCM family-owned stores (46%) and TCM chain stores (43%) than in other locations (χ^2^(1, N = 403) = 2.90, p <0.04; χ^2^(1, N = 403) = 353.73, p <0.001 respectively; Fig 2C). No respondents purchased saiga products online, and in fact, there were only 12 reports of online purchases for any treatment type purchased for oneself or others. When asked what other treatment types they used, self-users were significantly less likely than non-users to also use western medicine or “Other” medicines (ß = -1.330, z = -8.879, p <0.001; ß = -1.513, z = -9.138, p <0.001 respectively).

### Overall user trends

**Fig 3:**
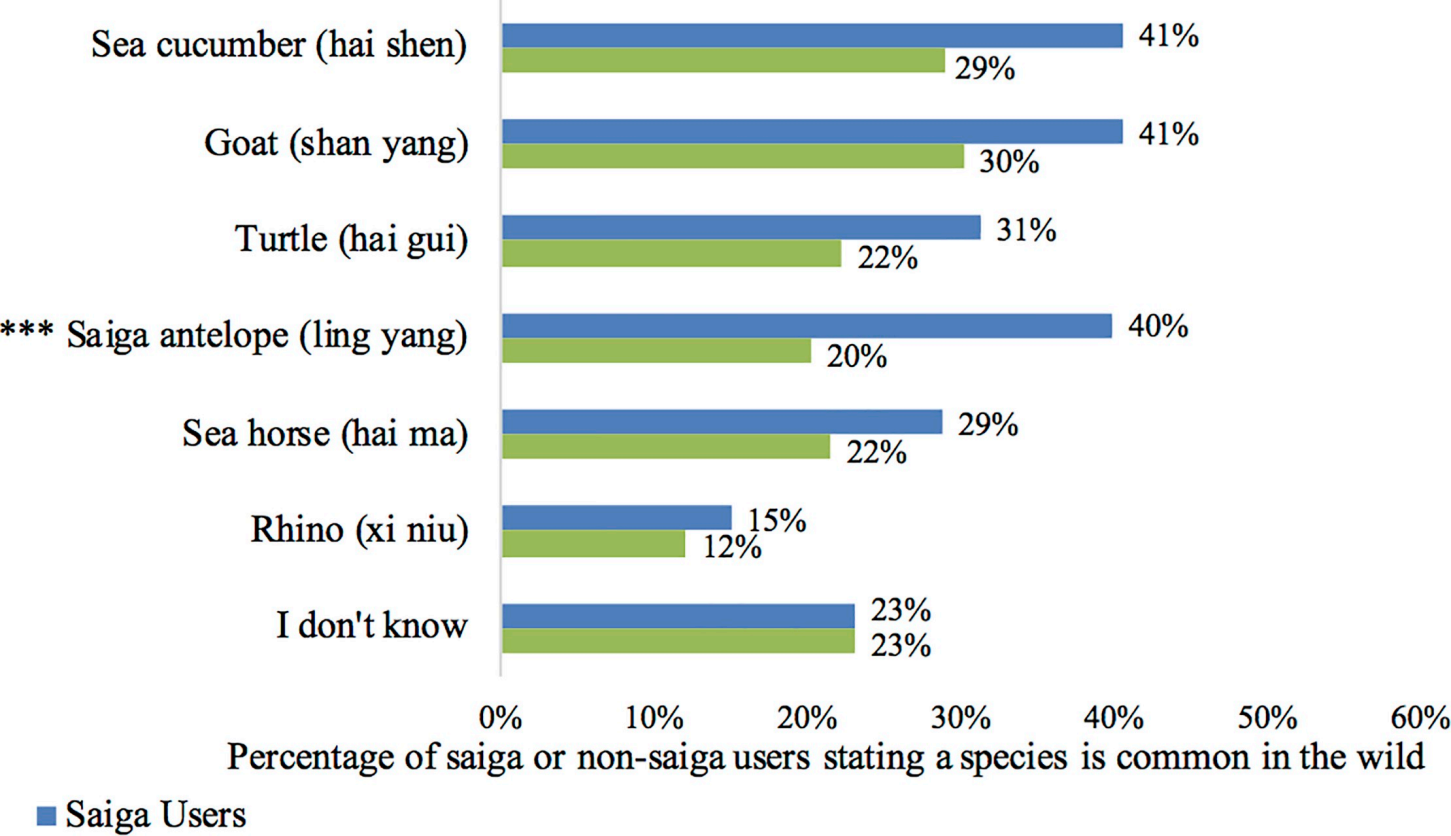
Perceived commonness of species in the wild. *** indicates a statistically significant association between saiga users and perceiving that animal as common (p-value <0.001, Pearson’s Chi-squared test). Percentages out of 438 participants for saiga users and 1,856 for non-users.

Overall saiga users (i.e. all self-users and buyers) were significantly more likely to be surveyed in lower socio-economic locations than in other locations (Pearson's Chi-squared test, x^2^(1, *N* = 438) = 12.918, *p* <0.001). Finally, we assessed respondents’ perceptions of species used in TCM. We found that saiga users had a greater overall propensity than non-users for perceiving animals as common in the wild (ß = 0.023, z = 3.665, p <0.001). When looking specifically at each animal though, the difference between saiga and non-saiga user perception was significant only for saiga (Pearson's Chi-squared test, x^2^(1, *N* = 438) = 38.56, *p* <0.001) (Fig 3).

## Discussion

The main saiga users in our sample are middle-aged Chinese Singaporeans who identify as Buddhist or Taoist. The relatively high prevalence of usage (19%) is evidence that saiga is a commonly used TCM product. This level of consumption has been seen for other TCM products derived from wildlife, such as bear bile consumers in China [34], and saiga horn specifically has been previously observed as a common product in TCM markets in countries like China [35]. However, the usage level we found in Singapore was unexpected given the low level of legal trade into Singapore reported to CITES over the last few years [20]. This may suggest that the stockpile of horns which was reported as being in Singapore at the time of the species being listed on CITES may be decreasing. The strong influence of family and TCM shopkeepers on a consumer’s choice to use saiga horn is also particularly noteworthy for tailoring future demand reduction efforts that may want to employ social or professional influencers [36,37].

### Consumer groups

When comparing results with our *a priori* hypotheses, we found expected associations between saiga products and age and religion across consumer sub-groups. In contrast to the preliminary findings of [21], we hypothesised that saiga product use would increase with age, as general perceptions in Singapore are that TCM use is more popular with older generations. A relationship with age was supported by the significant negative association between younger respondents and saiga product use, contrasting to the significant positive association between middle-aged (though not elderly) respondents and saiga product use.

The strong associations of saiga product use with Buddhism and Taoism were expected given the cultural overlaps between TCM and these religions [31]. This trend was highlighted when comparing buyers of saiga products to buyers of any treatment (including western treatments), who were not more likely to be associated with any religion. The strong negative association between Christianity, particularly Catholicism, and saiga self-use was unexpected. The robust positive correlations between saiga horn consumption and Buddhism and Taoism have important implications for future intervention approaches. Researchers can thus consider faith-based strategies that are culturally relevant and respectful, as well as have a greater potential for successful uptake. Buddhism is one of the most environmentally benevolent religions [38] and Buddhist monasteries have had active roles in past conservation programs [39]. As such, behavioural interventions that work in collaboration with Buddhist temples, for example, to highlight how the use of saiga products links to wildlife damage, could potentially be effective in our study population. Utilising a faith-based approach to addressing wildlife trade was innovatively implemented among Muslim communities in Indonesia through the issuing of a Fatwa (Islamic law) against wildlife trafficking [40].

Buyers of any treatment, including saiga products, were most likely to be middle-aged women. Women’s role in making health decisions for others within their interpersonal network is well documented [41] and as such, our findings confirmed our hypothesis on gender for buyers. A 2006 study found Singaporean wives, more than husbands, were the ones to purchase over-the-counter medicine for their family unit [31]. Additionally in our survey, for those purchasing any treatment, respondents with higher education were more likely to purchase products for others, but this trend was not observed for saiga buyers specifically.

We hypothesized that education level may impact saiga product consumption. Education appeared in the top models for the users and self-users, however the associations were not significant and the variable's importance was <0.8. We also hypothesised that the number of generations a respondent's family had been living in Singapore might be negatively correlated with saiga product usage, on the assumption that people more recently arrived from China may have a stronger attachment to TCM, but this was not supported by the data. Similarly, we anticipated that TCM would be used more often by lower income people, since some forms have been referenced as cheaper than biomedical alternatives [24], but this was not fully supported by our results. The same 2005 study also did not find income to influence complementary medicine usage in Singapore (the most common of which is TCM) [24]. Despite having no significant coefficient though, income did appear strongly in the top model for buyers, with a variable importance of 0.82, suggesting that it may be associated with this behaviour. A large percentage (39%) of respondents chose to not state their income level, so it is possible that correlations with income do exist. Further supporting our original hypothesis, we found that the proportion of saiga users in our sample was highest in lower socio-economic survey locations. More research is thus needed on this point to better understand possible trends.

Descriptive results highlighted to us that even though particular demographic characteristics, like identifying as Buddhist, may make an individual significantly more likely to be a saiga user, there is a lot of demographic variation within the saiga user group. In fact, for the young age group, “No religion” was the second most common religious affiliation of saiga users. Saiga product use was seen across a range of demographic groups, be it religious, educational, or dialect group; suggesting that saiga use is prevalent across the sample.

### Preferences and motivations

There were interesting product-specific differences seen in the data. Saiga horn shavings, which were significantly more popular among middle-aged women, are a relatively traditional form of the product (one step beyond purchasing the whole horn and shaving it yourself). Shavings require an individual to boil them at home, coupled with a prescribed set of herbs. Some shopkeepers in our study area recommended boiling for two to four hours before consumption. Saiga horn cooling water products, however, can be immediately consumed upon purchase. They are therefore a more modern form of the product, that has been pre-boiled for a consumer, and do not require any effort. They are also often sold chilled, which likely adds to their desirability to a consumer who is feeling heaty or feverish. Cooling water self-users were significantly more likely to be male. Strong gender differences in the consumption of wildlife products has been seen for other species as well. For instance, in Vietnam, it has been suggested that women may be the main purchasers of rhino horn, but wealthy middle-aged men may be the main end-users of the product [42]. Thus, our results suggest that demand reduction efforts might consider tailoring by gender if focusing on one of these product forms.

We were surprised to see the lack of online purchases for any treatment type, given the high prevalence of online shopping in Singapore, and the rise in online sales of both biomedical and traditional medicines in Singapore stated in the Health Science Authority of Singapore text [43]. Clearly more research is needed on online purchase behaviour of over-the-counter medicines in Singapore that may contain sensitive species, in order to better monitor and regulate potentially unsustainable usage patterns.

Regarding motivations for consumption, the highly reported "Someone recommended it to me" answer echoes the well-documented influence of interpersonal communication on human behaviour, including health choices [44]. Specifically within Singapore, Chang et al. found that Chinese Singaporean women cited their spouses, parents, and peers as key ongoing influences in their health decisions [41]. The particular impact of familial influence was seen in "Family" being the most reported recommender for saiga product self-usage among our respondents. In addition to recent direct recommendation of a given health product to a respondent, 'recommendation' could well have occurred in a broad sense many years prior, particularly during a respondent's childhood. The effect of caregiver health decisions and opinions has been shown repeatedly to impact child health choices in areas like vaccination records [45], and cold medication use [46], with many of these influences stretching through adulthood, as exemplified in food pallet conditioning [47,48], and alcohol use [49]. The second most-reported recommender, a “TCM shopkeeper”, is likely to be due to saiga products being most often purchased in TCM shops, where consumers frequently seek the advice of the shopkeeper [29], similar to a biomedical pharmacist [37]. The use of pharmacies for delivering public health interventions is well documented and could provide good insights into possible saiga interventions utilising TCM shopkeepers’ influence [50].

"It works" was the most common reason for using saiga product among our respondents. This perception of efficacy could be due to many factors, such as an individual's personal past experience of using saiga products, a restatement of effects communicated to them by others, the observation of others using it with positive results, or possibly some combination of these factors. It is therefore likely that the top two answers given by respondents as to why they use saiga are highly intertwined, and disentangling them will require further research.

It was interesting to note the difference between saiga users and non-users in their perception of wildlife commonness. Whether perception leads to usage, or usage is justified by perception, or an entirely different cause affects both variables, is unknown. For instance, it is possible TCM consumers are used to seeing these products in stores and therefore are more likely to perceive the animals as common. Additional work to elucidate perceptions of commonness is important in order to develop effective messaging for reduction of demand for species perceived as common but which are actually threatened.

### Future research implications

Our survey was conservative in that it asked respondents about their most commonly used products, so we feel confident that *high-level saiga users* compose 19% of the sample, and that overall saiga product use (including less frequent users) is higher. Given our findings, it may be possible that a similar proportion of the 2.9 million Chinese Singaporean residents buy or use saiga products frequently for themselves or others [51]. There are likely also saiga product users among the many non-resident Chinese living in Singapore, and among non-Chinese Singaporeans. This figure cannot be easily translated into a number of horns (or animals used) per year, however, because the amount of horn per dose is not known. This amount is likely to be relatively small, but varies between product types and even between stores which prepare their own products. Some products marketed as saiga/antelope do not contain any saiga horn, though the degree to which this occurs is also not verified. Saiga horn use in Singapore is nonetheless extensive, and non-negligible. This study paves the way for a grounded behavioural change intervention with a clear target audience and focus. We further hope that this work gives useful guidance on research approaches for understanding the use of saiga products in other countries, as well as for other unsustainably traded species that are perceived as commonplace by consumers.

## Supporting information

S1 Table Consumer survey. (PDF)

S2 Table Survey locations. (PDF)

S3 Table Raw data. (XLSX)

S1 File Survey size power calculation. (PDF)

S2 File Buyers of any treatment. (PDF)
