# Correction: Saiga horn user characteristics, motivations, and purchasing behaviour in Singapore

**DOI:** 10.1371/journal.pone.0226721

**Published:** 2019-12-12

**Authors:** Hunter Doughty, Diogo Veríssimo, Regina Chun Qi Tan, Janice Ser Huay Lee, L Roman Carrasco, Kathryn Oliver, E. J. Milner-Gulland

The percentages for the “Non-Saiga Users” group are incorrect in Fig 3. The authors have provided a corrected version here.

**Fig 3:**
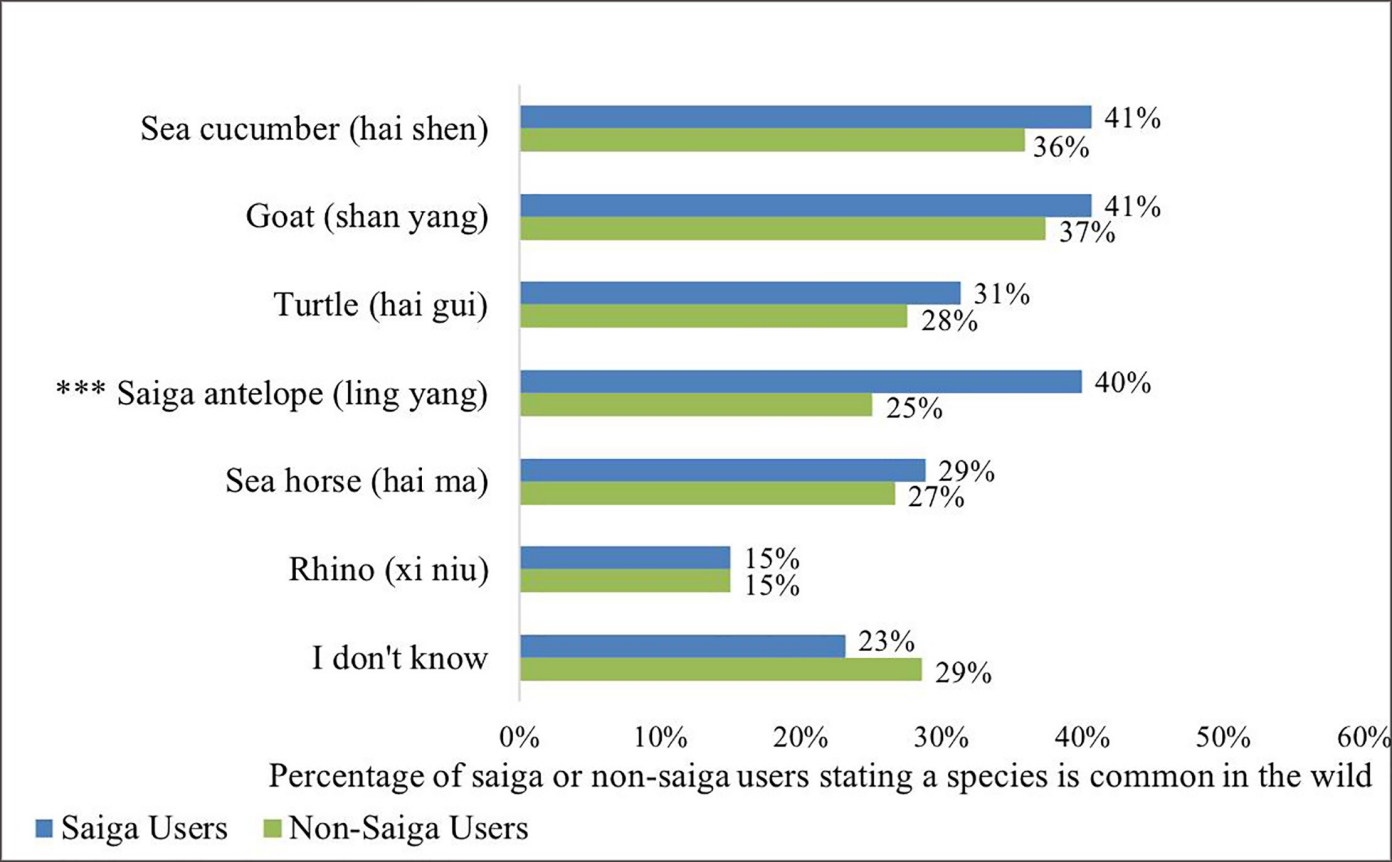
Perceived commonness of species in the wild. *** indicates a statistically significant association between saiga users and perceiving that animal as common (p-value <0.001, Pearson’s Chi-squared test). Percentages out of 438 participants for saiga users and 1,856 for non-users.
