# Supplementary material for: Saiga horn user characteristics, motivations, and purchasing behaviour in Singapore
Source: PLoS One. 2019 Sep 10;14(9):e0222038. doi: 10.1371/journal.pone.0222038 (PMC6736248; doi:10.1371/journal.pone.0222038)
Supplement: S1 Table — (PDF) [file pone.0222038.s001.pdf]

Supporting Information

Saiga horn user characteristics, motivations, and purchasing behaviour in Singapore

Doughty et al. 2019

S1 Table: Consumer Survey

Questions were organized into a decision-tree style format, allowing respondents to answer general questions first, and only be asked further questions as they were applicable to them.

CONSUMER SURVEY

SAIGA HORN AS A TRADITIONAL MEDICINE IN SINGAPORE

The survey used in this study was a decision tree style format in which respondents were asked certain subsequent questions depending on the answers they provided. For brevity, each specific question and answer set is only shown here *once*. In other words, only the first time a question and answer set is asked, the question and answer set is written out in full.

**Question Number**		**Question Number that a respondent would proceed to, depending on the answer selected; if no number is listed here then the immediately proceeding question would follow.**
1	The following questions address treatments for heatiness.<br><br>Heatiness is defined as having a body temperature below 38°C but having symptoms such as: sore throat, nasal congestion, headache, fatigue, and cough.	
1a	If you are feeling heaty, which of the following treatment(s) do you use most often? [Select all that apply]	
	Western product (e.g. isotonic drinks)	2
	Herbal traditional product (e.g. chrysanthemum tea)	4
	Animal traditional medicine product (e.g. saiga antelope [ling yang])	6
	Other	1b
1b	What other heaty treatments do you use most often?	
2	WHEN TREATING HEATINESS WITH TRADITIONAL ANIMAL PRODUCTS:	
2a	Which type of animal products do you use most often? [Select all that apply]	
	Goat (shan yang)	
	Saiga antelope (ling yang)	
	Sheep (mian yang)	
	Buffalo (shui niu)	
	Other	
2b	What form of treatment do you use most often?	
	Cooling water	

	Pills/capsules	
	Fever patch	
	Shavings of animal horn	
	Whole animal horn	
2c	Who purchases this treatment for you?	
	Myself	
	A parent/A grandparent	
	A spouse	
	My child	
	Other	
2d	Where do you normally shop for this treatment? [Select all that apply]	
	Pharmacy	
	Traditional medicine chain store	
	Traditional medicine family-owned store	
	Family-owned provision shop	
	Supermarket	
	Online	
	Other	
2e	Why do you prefer using this treatment? [Select all that apply]	
	It works	
	Someone recommended it to me	3
	It's affordable	
	It's convenient	
	I don't know	
	Other	3b
3	WHEN TREATING HEATINESS WITH TRADITIONAL ANIMAL PRODUCTS:	
3a	Who recommended this treatment to you? [Select all that apply]	
	Western doctor	
	Traditional medicine practitioner	
	Pharmacist	
	Traditional medicine shopkeeper	
	Family	
	Friends	
	Other	
3b	What other reason makes you prefer this treatment?	

4	WHEN TREATING HEATINESS WITH WESTERN MEDICINE PRODUCTS:	
4a	What form of treatment do you use most often?	
	Cooling water (such as isotonic drinks)	
	Pills/capsules	
	Fever patch	
	Fever syrup	
	Other	
4b	Who purchases this treatment for you?	
4c	Where do you normally shop for this treatment? [Select all that apply]	
4d	Why do you prefer using this treatment? [Select all that apply]	
5	WHEN TREATING HEATINESS WITH WESTERN MEDICINE PRODUCTS:	
5a	What other reason makes you prefer this treatment?	
5b	Who recommended this treatment to you? [Select all that apply]	
6	WHEN TREATING HEATINESS WITH TRADTIONAL HERBAL PRODUCTS:	
6a	What form of treatment do you use most often?	
	Cooling water (such as chrysanthemum tea)	
	Pills/capsules	
	Fever patch	
	Fresh or dried herbs	
	Other	
6b	Who purchases this treatment for you?	
6c	Where do you normally shop for this treatment? [Select all that apply]	
6d	Why do you prefer using this treatment? [Select all that apply]	
7	WHEN TREATING HEATINESS WITH TRADTIONAL HERBAL PRODUCTS:	
7a	What other reason makes you prefer this treatment?	
7b	Who recommended this treatment to you? [Select all that apply]	
8	Do you purchase heatiness medication for others? If so who? [Select all that apply]	
	A child (0 - 12 years old)	9
	A teenager (13 - 20 years old)	10 (not shown)
	An adult (21 years and above)	8a; 11 (not shown)
	I do not purchase medication for anyone else	
8a	How are you related to this/these adults? [Select all that apply]	
	My older relative	
	My younger relative	

	My spouse	
	My child	
	My friend	
	Other	
9	Do you buy a different type of heatiness medication for children (0 - 12 years old) than you use for yourself?	Depending on 8 answer, respondent will be asked about child, teen, and/or other adult purchases
	Yes	
	No	9a
	It depends	9a
9a	When buying heatiness medication for a child, which of the following treatment(s) do you use most often? [Select all that apply]	Depending on 9 answer, respondent will be asked to expand on their decision using the same decision tree as the 'self use' questions
12	If you are experiencing only slight heat-related discomfort, such as after exercising or being in the sun, would you use the same treatment as you use for heatiness?	
	Yes	
	No	
	It depends	
12a	Please specify what cooling treatment you would choose.	
13	The following questions address treatments for fever.<br><br>Fever is defined as having a body temperature of 38°C and above.	The entire decision tree asked of heatiness medication preferences is repeated for fever medication preferences
13a	If you get a fever, which of the following treatment(s) do you use most often? [Select all that apply]	
	Western medicine product (e.g. *Panadol*)	
	Herbal traditional product (e.g. chrysanthemum tea)	
	Animal traditional medicine product (e.g. saiga antelope [ling yang])	
	Other	
14	Which of these animals used in traditional medicine products are common in the wild? [Select all that apply]	All respondents are asked to answer questions 14 - 15f
	Sea cucumber (hai shen)	

	Goat (shan yang)
	Turtle (hai gui)
	Saiga antelope (ling yang)
	Sea horse (hai ma)
	Rhino (xi niu)
	I don't know
**15**	What is your gender?
	Male
	Female
	Prefer not to say
**15a**	What is your age?
	18 - 35 years
	36 - 59 years
	≥ 60 years
	Prefer not to say
**15b**	What is the dialect group of your family?
	Hokkiens
	Teochews
	Cantonese
	Hakkas
	Hainanese
	Other
	Prefer not to say
**15b.2**	Please specify the dialect group of your family.
**15c**	What religion do you identify yourself most with?
	Buddhist
	Muslim
	Taoist
	Catholic
	Hindu
	Christian (other)
	Other
	I do not identify with a religion
	Prefer not to say
**15c.2**	Please specify the religion you most identify with.
**15d**	What generation of Singaporean are you?

	First<br>Second<br>Third<br>More than third<br>Prefer not to say	
**15e**	What is your highest level of education?	
	Pre-primary or primary school<br>Secondary school<br>ITE<br>Polytechnic<br>Junior college<br>Accredited diploma<br>University<br>Masters or PhD<br>Prefer not to say	
**15f**	What is the approximate total MONTHLY income for your household?	
	Less than $5000<br>$5001 to $15,000<br>$15,001 to $25,000<br>$25,001 to $35,000<br>$35,001 and higher<br>Prefer not to say	
