# Supplementary material for: Saiga horn user characteristics, motivations, and purchasing behaviour in Singapore
Source: PLoS One. 2019 Sep 10;14(9):e0222038. doi: 10.1371/journal.pone.0222038 (PMC6736248; doi:10.1371/journal.pone.0222038)
Supplement: S2 Table — (PDF) [file pone.0222038.s002.pdf]

Supporting Information

Saiga horn user characteristics, motivations, and purchasing behaviour in Singapore

Doughty et al. 2019

S2 Table: Survey Locations

Within each Planning Area, we identified four locations that attract varying socio-economic levels. Two 'low' locations were chosen for each Planning Area as these tended to be smaller in scale and traffic.

Planning Areas with the largest population of Chinese		Locations within each Planning Area whose clientele include members of the below income levels (all locations likely have clientele from *all* income levels).			
**Planning Area**	**Number of Chinese**	**Includes High Income**	**Includes Medium Income**	**Includes Low Income**	
		**Location 1**	**Location 1**	**Location 1**	**Location 2**
Bedok	208,880	Siglap Centre	Bedok Mall	Bedok South Food Centre	The Market Place @58
Jurong West	190,240	Jurong Point	Gek Poh Shopping Centre	Boon Lay Shopping Centre	Taman Jurong Shopping Centre
Hougang	181,700	Heartland Mall	Hougang Mall	Hougang Green Shopping Mall	Hougang 105 Hainanese Village Centre
Tampines	175,470	Tampines Mall	Eastpoint Mall	Hawker Centre 201	Prime Supermarket (Tampines Street 81)
Sengkang	160,590	Rivervale Mall	Compass One	The Seletar Mall	Kopitiam @ Block 275 D Compassvale Link
**Total in top 5 Planning Areas**		916,880			
**Total in Singapore**		2,900,010			
