# Supplementary material for: Saiga horn user characteristics, motivations, and purchasing behaviour in Singapore
Source: PLoS One. 2019 Sep 10;14(9):e0222038. doi: 10.1371/journal.pone.0222038 (PMC6736248; doi:10.1371/journal.pone.0222038)
Supplement: S1 File — (PDF) [file pone.0222038.s004.pdf]

Supporting Information

Saiga horn user characteristics, motivations, and purchasing behaviour in Singapore

Doughty et al. 2019

S1 File: Survey Size Power Calculation

Using nested analyses as a gauge, we conducted a power calculation for performing a three-tiered nested proportion statistical test with three of our most integral questions.

Example three nested proportion questions we hoped to analyse:
  Highest level question: Saiga use -> 13% for usage -> 202.5/0.13 = 1558ppl
  Second level question: Reasons for use -> est40% for recommendation -> 81/0.4 = 202.5ppl
  Third level question: Recommenders for use -> est30% for family -> 81ppl

  Margin of error = 10% at the lowest level

Equation Used:
  n = N*X / (X + N – 1),
"where, X = Zα/22 ¬*p*(1-p) / MOE2, and Zα/2 is the critical value of the Normal distribution at α/2 (e.g. for a confidence level of 95%, α is 0.05 and the critical value is 1.96), MOE is the margin of error, p is the sample proportion, and N is the population size." (https://bit.ly/2qMON7Z)
