# Supplementary material for: Saiga horn user characteristics, motivations, and purchasing behaviour in Singapore
Source: PLoS One. 2019 Sep 10;14(9):e0222038. doi: 10.1371/journal.pone.0222038 (PMC6736248; doi:10.1371/journal.pone.0222038)
Supplement: S2 File — (PDF) [file pone.0222038.s005.pdf]

Supporting Information

Saiga horn user characteristics, motivations, and purchasing behaviour in Singapore

Doughty et al. 2019

S2 File: Buyers of Any Treatment

When comparing saiga buyers to buyers of any treatment type for others we found that the two groups were fairly similar in gender and age, but that for this larger buyer group, education was positively related to buying for others, and religion was not a variable of importance. Buyers for Others was composed of participants who buy any treatment type for others to treat heatiness and/or fever (1196 respondents). Statistical Analysis: GLM dredge and average, with sum contrasts applied.

A priori hypotheses:

		Hypothesis
Variable	*Effect Y/N*	*Direction of Effect*
Age	Y	Middle-age more likely to buy for others
Education	N	No difference between education levels
Religion	N	No difference between religions
Gender	Y	Females more likely to buy for others
Dialect	N	No difference between dialect groups
Income	N	No difference between income levels
Generation	N	No difference between generations

Model selection table:

	(Intrc)	age	education	gender	generation	df	logLik	AICc	delta	weight
30	-0.1592	+	+	+	+	12	-1462.081	2948.3	0	0.858
14	-0.208	+	+	+		8	-1467.918	2951.9	3.6	0.142

Relative variable importance:

	age	education	gender	generation
Importance:	1	1	1	0.86

Model-averaged coefficients:

(full average)

	Estimate	Std. Error	Adjusted SE	z value	Pr(>|z|)	P-value•	Likelihood ^
(Intercept)	-0.16611	0.07641	0.07645	2.173	0.0298	*	
young	-0.66039	0.07525	0.07529	8.771	< 2e-16	***	negative
mid-age	0.68968	0.06431	0.06434	10.719	< 2e-16	***	positive
old	-0.02929	0.07747	0.07751	0.378	0.7056		neutral
primary	-0.24929	0.11241	0.11247	2.216	0.0267	*	negative
secondary-ite	0.07054	0.08957	0.08962	0.787	0.4312		neutral
preuni-postsec	0.09932	0.09399	0.09404	1.056	0.29091		neutral
uni-grad	0.58744	0.09979	0.09984	5.884	< 2e-16	***	positive
unknown edu	-0.50801	0.16583	0.16592	3.062	0.0022	**	negative
female	0.36633	0.08888	0.08893	4.119	3.80E-05	***	positive
male	-0.36633	0.08888	0.08893	4.119	3.80E-05	***	negative
first	-0.02194	0.08805	0.0881	0.249	0.8034		neutral
second	-0.11389	0.08785	0.08789	1.296	0.195		neutral
third	0.13688	0.10411	0.10415	1.314	0.18873		neutral
more than third	0.26325	0.17849	0.17855	1.474	0.1404		neutral
unknown gen	-0.26431	0.17674	0.1768	1.495	0.1349		neutral

•Signif. codes: 0 ‘***’ 0.001 ‘**’ 0.01 ‘*’ 0.05 ‘.’ 0.1 ‘ ’ 1

^Direction of likelihood of being a saiga user at this level of confidence
